# 4109 Acceptability of a Tenofovir Disoproxil Fumarate Intravaginal Ring for Human Immunodeficiency Virus Pre-Exposure Prophylaxis Among Sexually Active Women

**DOI:** 10.1017/cts.2020.105

**Published:** 2020-07-29

**Authors:** April Dobkin, Rebecca Barnett, Jessica McWalters, Laurie L. Ray, Lilia Espinoza, Aileen P. McGinn, Jessica M. Atrio, Marla J. Keller

**Affiliations:** 1 Albert Einstein College of Medicine

## Abstract

OBJECTIVES/GOALS: Vaginal ring delivery of antiretroviral drugs may provide protection against acquisition of HIV-1 when used as pre-exposure prophylaxis. As part of a randomized placebo-controlled safety trial of a tenofovir disoproxil fumarate (TDF) intravaginal ring (IVR), we assessed product acceptability through surveys of 17 women after continuous ring use. METHODS/STUDY POPULATION: Sexually active, HIV negative women between the ages of 18 and 45 were enrolled to investigate the safety and pharmacokinetics of three months of continuous TDF IVR use. The study was designed to include 40 US participants randomly assigned (3:1) to a TDF or placebo IVR. Twelve were randomized to TDF and five were assigned to the placebo group before the study was electively discontinued due to development of vaginal ulcerations in eight women in the TDF group. Acceptability data regarding TDF and placebo ring use was gathered via self-administered, computer-based questionnaires at the one- and three-month study visits. Participants were asked about overall attitudes and feelings regarding the TDF and placebo IVR, vaginal changes associated with ring use, and their experiences with ring use during menses and with sex. RESULTS/ANTICIPATED RESULTS: The mean age of participants was 30 years (range 18 - 42). Sixteen of 17 (94%) participants completed all study questions at both visits. When asked about ring likeability at one-month, 12 of 16 (75%) women reported overall liking the ring, including 5 of 8 (63%) who developed ulcerations. Vaginal changes described during ring use included 8 participants who indicated that the “vagina was wetter” and 2 who reported that the “vagina was drier.” Additionally, 10 of 12 (83%) who had their period during the first month of the study were not bothered by ring use during menses, and 11 of 16 (69%) stated that the ring was not bothersome with use during sex. When asked at the three-month visit, most reported that they would prefer to wear the ring rather than use a condom during sex, however, condom use was low at baseline in this population. DISCUSSION/SIGNIFICANCE OF IMPACT: Despite unanticipated ulcers, the IVRs were acceptable, especially when used with menses and during sex. Regardless of the group assigned or vaginal changes experienced, and even amongst those who developed ulcerations, the women had positive attitudes towards the ring, which is promising for future use of vaginal rings as a method for HIV prevention.

